# Mucinous Cystadenoma Arising in a Uterine Isthmocele: A Case Report

**DOI:** 10.1055/s-0043-1770090

**Published:** 2023-07-21

**Authors:** Jesus Paula Carvalho, Alexandre Silva e Silva, Rodolpho Truffa Kleine, Marília Albanezi Bertolazzi, Rodrigo Pinto Fernandes, Filomena Marino Carvalho

**Affiliations:** 1 Faculdade de Medicina, Universidade de São Paulo, São Paulo, SP, Brazil; 2 Instituto do Câncer do Estado de São Paulo, Faculdade de Medicina, Universidade de São Paulo, São Paulo, SP, Brazil

**Keywords:** isthmocele, niche, diverticulum, deficient uterine scar, scar pouch, mucinous cystadenoma, istmocele, nicho, divertículo, cicatriz uterina deficient, bolsa cicatricial, cistadenoma mucinoso

## Abstract

Isthmocele is a discontinuation of the myometrium at the uterine scar site in a patient with a previous cesarian section (CS). The cause of isthmocele appears to be multifactorial. Poor surgical technique, low incision location, uterine retroflection, obesity, smoking, inadequate healing of scars, and maternal age are possible related factors. Most patients with this condition are asymptomatic. However, women can present with postmenstrual bleeding, pelvic pain, subfertility, dysmenorrhea, infertility, and scar abscess. Brazil has one of the world's highest cesarean section rates. One of the consequences of the rising rate of CS is the isthmocele, an emerging female health problem. Here we report a case of mucinous cystadenoma arising in a uterine isthmocele, a complication, as far as we could investigate, not yet described in the literature.

## Resumo

Istmocele é a descontinuidade do miométrio no local da cicatriz uterina em paciente com cesariana anterior. A causa da istmocele parece ser multifatorial. Má técnica cirúrgica, baixa localização da incisão, retroflexão uterina, obesidade, tabagismo, cicatrização inadequada de cicatrizes e idade materna são possíveis fatores relacionados. A maioria dos pacientes com esta condição é assintomática. No entanto, as mulheres podem apresentar sangramento pós-menstrual, dor pélvica, subfertilidade, dismenorreia, infertilidade e abscesso cicatricial. O Brasil tem uma das maiores taxas de cesariana do mundo. Uma das consequências da taxa crescente de cesarianas é a istmocele, um problema emergente de saúde feminina. Aqui relatamos um caso de cistoadenoma mucinoso originado em uma istmocele uterina, uma complicação ainda não descrita, até onde pudemos investigar.

## Introduction

Isthmocele is a cesarean scar defect, also named niche, diverticulum, deficient uterine scar, or scar pouch, defined as a discontinuation of the myometrium at the uterine scar site. 1 Radiologists define it as an indentation seen at the cesarean scar site with a depth of at least 2 mm at ultrasound or magnetic resonance imaging. 2 The standard diagnostic procedure for identifying isthmoceles is transvaginal sonography (TVS), and the prevalence ranges from 6.9% to 64.5%. 3 The cause of isthmocele appears to be multifactorial. Poor surgical technique, low incision location, uterine retroflection, obesity, smoking, inadequate healing of scars, and maternal age are possible related factors. Most patients with this condition are asymptomatic, although some of them can present postmenstrual bleeding, pelvic pain, subfertility, dysmenorrhea, infertility, and scar abscess. 2 4 One case of high-grade endometrial stromal sarcoma 4 and one of endometrioid carcinoma 5 originating in the scar has already been described. We have not found any description of mucinous cystadenoma or other tumors to date. Although the occurrence of neoplasm associated with isthmocele is an apparently rare event, considering the increase in the number of cesarean sections, we recommend special attention and investigation of symptoms in patients with previous cesarean.

## Case Description

**Fig. 1:**
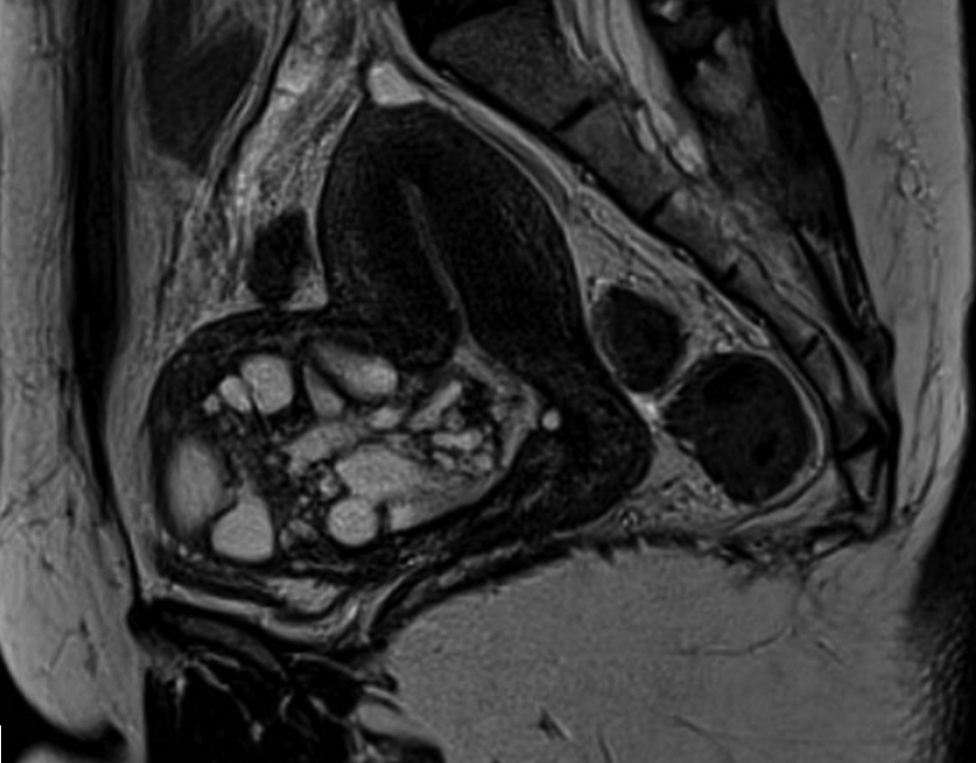
Magnetic Resonance Imaging (MRI) demonstrating a bulky isthmocele at cesarean section scar with several cysts inside.

A 49-year-old woman presented with a history of progressive vaginal fluid discharge of 3 months duration and a palpable pelvic mass. She was submitted to a cesarian section twelve years ago, followed by a history of infertility. The vaginal discharge was irregular, sometimes it happened spontaneously, sometimes during intercourse. She had no history of alcohol consumption or cigarette smoking. Her body mass index (BMI) was 26 kg/m2. A vaginal examination revealed a normal cervix and a heavy mucinous fluid discharge during the examination. Serum tumor marker CA125 levels were normal (CA125; 18 U/mL). Magnetic Resonance Imaging (MRI) demonstrated the uterus in anteversion, measuring 9.2 × 5.8 × 4.3 cm, with a volume of 119.3 cm ^3^ . There was a bulky multiloculated cyst measuring 7.5 × 7.0 × 6.6 cm (volume of 180.1 cm ^3^ ) at the level of cesarean scar in the anterior isthmocorporal uterine region ( Fig. 1 ). This complex isthmocele was filled with liquid content with a thick component and communicated with the uterine cavity in an extension of 2.5 cm. Both ovaries were normal.

**Fig. 2:**
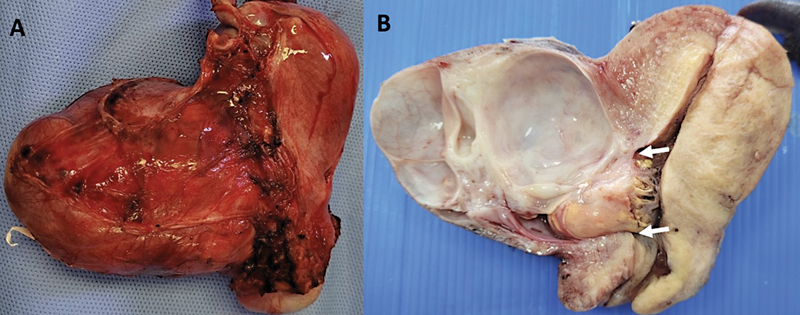
Surgical specimen showing a multiloculated cystic mass on the anterior uterine wall, in isthmocele associated with cesarean section scar: A) external view, B) uterine sagittal section showing communication of cystadenoma with uterine cavity (arrows).

**Fig. 3:**
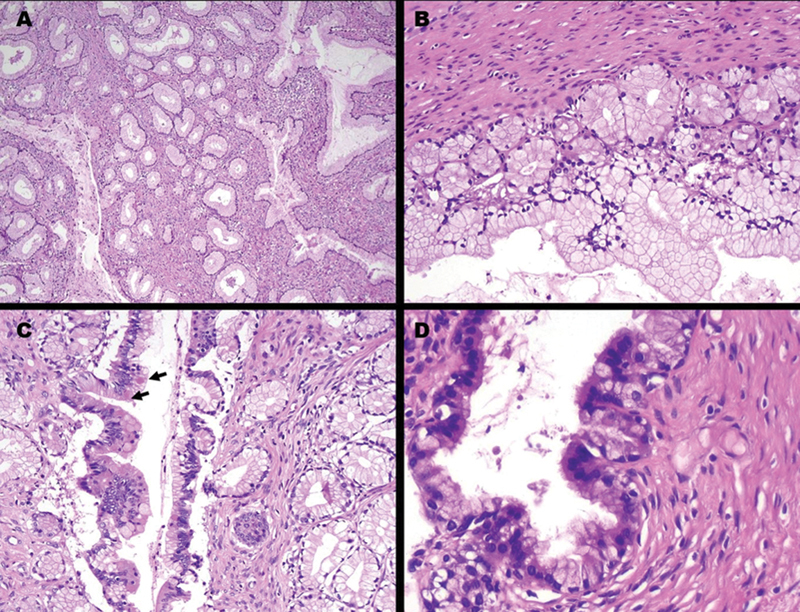
Mucinous cystadenoma in isthmocele A) densely packet mucinous glands B) detail of gland grouping with mucinous pyloric and goblet cells surrounding cystic space C) area of mild cell proliferation, without atypia, with mucinous and endocrine cells (arrows) D) gland showing numerous ciliated tubal serous-like cells.

We performed an extra-fascial laparoscopic hysterectomy plus bilateral salpingectomy ( Fig. 2 ). Pathologic examination revealed a multiloculated cystic lesion in the isthmic uterine scar with two holes communicating the tumor with the endocervix. The cysts were lined by pyloric type mucinous epithelium with goblet cells and some endocrine cells and serous foci. Cysts were surrounded by densely packed round mucinous glands ( Fig. 3 ). The pathological diagnosis was mucinous cystadenoma in the uterine isthmocele. The patient recovered well in the postoperative period and remains under follow-up, asymptomatic, with normal activities for 2 months, including sexual activity.

## Discussion

There is a gradual increase in CS globally. The highest rate is in Latin America, with an estimated CS rate of 42.8 (37.6, 48.0) (95% CI). 6 Brazil has one of the world's highest cesarean section rates. 7 With this increase, a rise in the incidence of isthmocele is expected. Several complications are related to cesarean section scar and isthmocele. The incidence of cesarean scar ectopic pregnancies, for example, is ∼1 in 2000 pregnancies. 8 There is a strong association between abnormal uterine bleeding (AUB) and cesarean scar defects (CSDs). These patients experience the so-called prolonged menstruation and early-cycle intermenstrual bleeding. 9 Pre-labor uterine rupture is another complication that may result in a severe risk of death.

Clinical and radiologic differential diagnosis may be challenging in some cases. Uterine parietal cysts with sometimes stale/bloody content look like retention cysts, but cannot completely rule out others diseases like tunnel clusters and cervical adenocarcinoma type gastric (former minimal deviation adenocarcinoma or adenoma malignum), that can present the same image aspects. Usually, the histologic findings from uterine specimens of isthmoceles are endocervical mucosa, with cystically dilated glands, and/or an atrophic or disorganized endometrial mucosa of lower uterine segment origin, showing variable regenerative epithelial atypia, fibroblastic stromal reaction, significant inflammation, and hemorrhage. 10

The association between isthmocele and benign/or malignant uterine tumor is unclear. Yi-Liang Lee et al. reported one case of high-grade endometrial stromal sarcoma in a 45-year-old woman who underwent hysteroscopic isthmoplasty. 4 Gorostidi and Rodriguez presented a case of endometrial carcinoma in a 44-year-old G1P1 woman that involved the isthmocele. 5 No description of mucinous neoplasms were found. This case presented the morphologic pattern seen in ovarian mucinous neoplasms, with gastrointestinal type epithelium, without cytologic atypia and, although showing architectural complexity, with no significant cellular proliferation. The morphological pattern of the mucinous cells was that seen in the lobular endocervical glandular hyperplasia, one of the lesions of the spectrum of gastric type epithelium lesion in uterine cervix. This type of lesion was even present in the endocervical canal. It is possible that the tumor originated in the endocervical mucosa included in the isthmocele or even in the endocervix itself, growing toward the isthmocele. We can hypothesized that the inflammatory microenvironment of the scar favored the neoplastic transformation of the included epithelium. The local inflammatory changes were also one of the discussed factors involved in the origin of the high-grade sarcoma in isthmocele described by Lee et al. 4

Treatment options are hysteroscopy-guided laparoscopic resection and repair of the cesarean scar, 11 hysteroscopic isthmoplasty, 12 or hysterectomy for those patients without reproductive desire. There is no data about the role between isthmocele and cervical neoplasia. As far as we know, this is the first case of a mucinous cystadenoma originating from isthmocele, However, considering the rising of this condition, it is prudent to investigate all cases of symptomatic isthmocele with pathological examination.

## Conclusion

We report what appears to be the first case of a mucinous neoplasm originating from a uterine isthmocele. Isthmocele is a uterine healing defect after a cesarean section. With the global increase in cesarean section rates in the world, and especially in Brazil, isthmocele and its complications may become a growing problem for women's health.
